# Ablation of Persistent Left Superior Vena Cava in Atrial Fibrillation Case

**DOI:** 10.1002/ccr3.9576

**Published:** 2024-11-29

**Authors:** Shuang Zhang, Mingxian Chen, Yichao Xiao, Lin Hu, Hanze Tang, Liyi Liao, Xuping Li

**Affiliations:** ^1^ Department of Cardiovascular Medicine The Second Xiangya Hospital of Central South University Changsha Hunan China

**Keywords:** ablaiton, arrhythmia, atrial fibrillation, persistent left superior vena cava

## Abstract

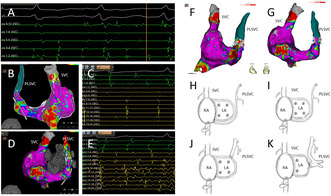

**Summary**

- Key clinical message
  ○ This case presents a patient with persistent atrial fibrillation (AF) that initial cryoablation followed by subsequent radiofrequency ablation identified the trigger for AF as persistent left superior vena cava (PLSVC).
  ○ Successful isolation of the PLSVC terminated AF, with no arrhythmia recurrence observed over a follow‐up period of more than 6 months.

## 1 Introduction

Persistent left superior vena cava (PLSVC) is a rare congenital vascular anomaly that occurs in approximately 0.3% of the general population and up to 4.4% of patients with congenital heart disease. It is formed by the persistence of the left cardinal vein, which typically regresses during embryological development [1]. While PLSVC itself is usually asymptomatic and often discovered incidentally, its presence can have significant implications for the electrophysiological properties of the heart.

The association of PLSVC with atrial fibrillation (AF) is particularly noteworthy. AF is the most common sustained cardiac arrhythmia and can lead to significant morbidity and mortality. The exact mechanism by which PLSVC contributes to AF is not fully understood, but it is believed that the abnormal electrical activity from the myocardial sleeves that extend into the PLSVC can act as triggers for AF. These myocardial sleeves can harbor ectopic foci that generate premature atrial contractions, which can initiate and perpetuate AF [2].

We report a case of persistent AF with a PLSVC. The patient underwent cryoballoon ablation, improving cardiac function. Six months later, the patient experienced paroxysmal AF and underwent radiofrequency ablation. Intracardiac echocardiography (ICE) and mapping confirmed PLSVC as the AF trigger. Isolation of the PLSVC successfully terminated AF. No AF episodes occurred during the 6‐month follow‐up. This case underscores the rarity of PLSVC as a trigger for AF and emphasizes the importance of thorough mapping and targeted ablation in managing such uncommon arrhythmogenic sources.

## 2 Case Presentation

### 2.1 Initial Presentation

A 58‐year‐old female patient was admitted to the hospital on February 22, 2023, due to recurrent palpitations accompanied by shortness of breath that she had experienced for more than 3 years. The patient has no comorbidities, including no history of hypertension, diabetes, or coronary artery disease.

### 2.2 Diagnostic Findings

The initial electrocardiogram (ECG) indicated AF, and an echocardiogram revealed enlargement of both the left and right atria, along with a decreased ejection fraction (EF) (left atrial size [LAS] 50 mm, left ventricular diameter [LVD] 55 mm, right atrial size [RAS] 39 mm, right ventricular diameter [RVD] 35 mm, EF 33%). The patient was diagnosed with persistent AF and treated with β‐blockers, ACE inhibitors, and SGLT2 inhibitors to manage symptoms and control heart rate and heart failure. Despite these treatments, there was no significant improvement, leading to the decision to pursue interventional therapy based on the patient's condition.

### 2.3 First Procedure

The patient underwent her first surgery with considerations for heart failure, as her EF had decreased, making it difficult for her to tolerate lengthy radiofrequency ablation procedures and intraprocedural saline infusion. Therefore, cryoablation was chosen for treatment. After the procedure, the patient maintained sinus rhythm for 6 months before experiencing a recurrence of paroxysmal AF.

### 2.4 Recurrence

On November 23, 2023, she was readmitted to the hospital due to persistent palpitations that had lasted for over 3 months. During these episodes, the ECG again showed AF. A follow‐up echocardiogram indicated a reduction in the size of both the left and right atria, and the EF had improved to normal (LAS 39 mm, LVD 45 mm, RAS 34 mm, RVD 29 mm, EF 61%).

### 2.5 Second Procedure

The patient was diagnosed with paroxysmal AF, and drug treatment showed no significant improvement. Consequently, a second interventional treatment was performed.

## 3 Methods (Electrophysiological Study and Ablation Procedure)

### 3.1 Discovery of Abnormal Structure

**FIGURE 1:**
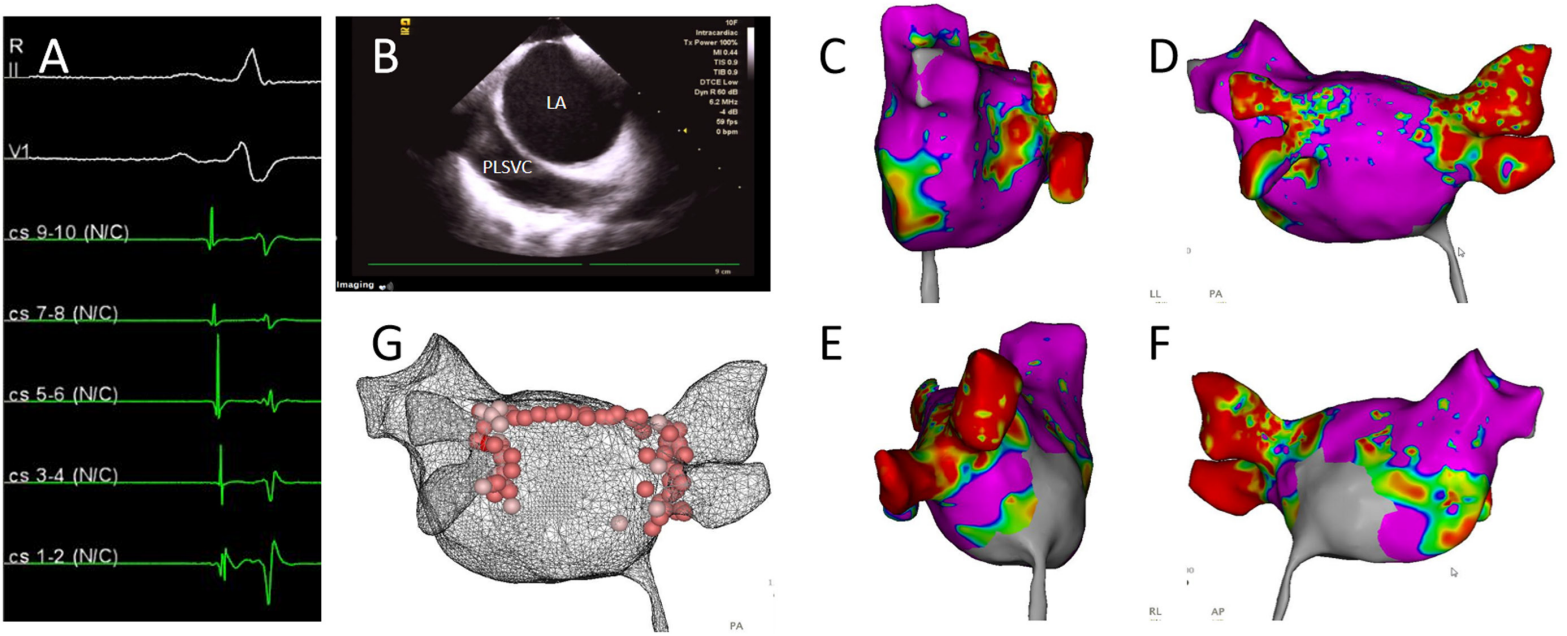
(A) The preoperative electrocardiogram indicates sinus rhythm. (B) Intracardiac echocardiography (ICE) shows an echolucent shadow alongside the left atrium (LA), consistent with a persistent left superior vena cava (PLSVC). (C) Three‐dimensional electroanatomical map of the left atrium in sinus rhythm, viewed from the left side. (D) Three‐dimensional electroanatomical map of the left atrium in sinus rhythm, viewed from the posterior–anterior perspective. (E) Three‐dimensional electroanatomical map of the left atrium in sinus rhythm, viewed from the right side. (F) Three‐dimensional electroanatomical map of the left atrium in sinus rhythm, viewed from the anterior–posterior perspective. (G) Ablation steps include gap ablation within the pulmonary veins, expansion of the ablation area, and linear ablation at the roof.

During the patient's second radiofrequency ablation procedure, pre‐operative assessments showed she was in sinus rhythm (Figure 1A). ICE revealed the presence of a PLSVC (Figure 1B). The intracardiac three‐dimensional mapping using the Carto 3 system demonstrated electrical reconnection in the left and right pulmonary veins (Figure 1C–F).

### 3.2 Ablation Steps

The ablation steps began with gap ablation within the pulmonary veins, followed by an expansion of the ablation area and linear ablation at the roof (Figure 1G).

### 3.3 Electrophysiological Examination

**FIGURE 2:**
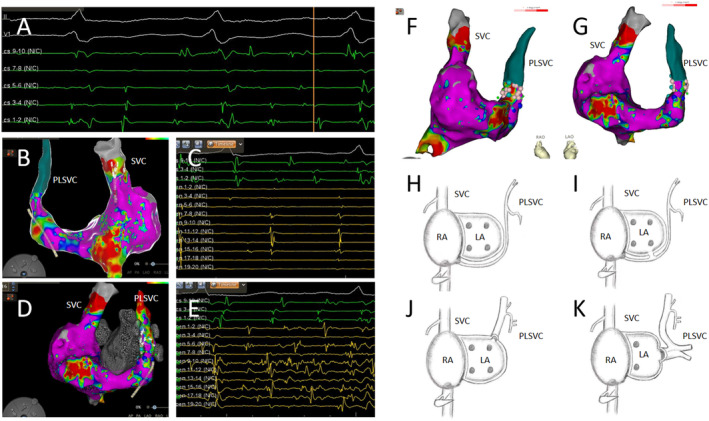
(A) Atrial fibrillation (AF) was induced using an S1S1 pacing protocol at 260 ms. (B) A three‐dimensional electroanatomical mapping of the right atrium shows the presence of a persistent left superior vena cava (PLSVC). The mapping electrode located within the superior vena cava (SVC) recorded potentials in the posterior–anterior view. (C) The mapping electrode (yellow) displays regular electrical potentials in the SVC, which are slower in frequency relative to those in the coronary sinus (CS) (green). (D)The three‐dimensional electroanatomical map of the right atrium with the mapping electrode positioned in the PLSVC records rapid and disorganized potentials in the anterior–posterior view. (E) The mapping electrode (yellow) in the PLSVC shows prolonged, fragmented, and regular potentials, which are faster than those in the CS (green). (F) A three‐dimensional model from the right anterior oblique perspective showing the ablation points (pink) at the proximal end of the PLSVC. (G) A three‐dimensional model from the left anterior oblique perspective also showing the ablation points (pink) at the proximal end of the PLSVC. (H–K) Three types of PLSVC configurations. (H) The PLSVC drains into the right atrium via the CS. (I) The PLSVC drains into the left atrium via the CS. (J) The PLSVC drains directly into the left atrium. (K) The PLSVC drains into the left atrium and is associated with pulmonary vein anomalies.

Post‐operatively, AF was induced using an S1S1 pacing protocol (Figure 2A), suggesting ectopic electrical activity originating from non‐pulmonary vein sources. Subsequently, a three‐dimensional electroanatomical mapping of the right atrium was performed, identifying the presence of a PLSVC (Figure 2B,D). The electrical potentials in the superior vena cava (SVC) were regular and slower compared to those in the coronary sinus (CS), indicating passive activation (Figure 2C). In contrast, the PLSVC exhibited prolonged, fragmented, and regular potentials, suggesting it as a driver of the AF (Figure 2E).

### 3.4 Ablation of PLSVC

Ablation was performed at the site of fragmented potentials within the PLSVC, with an ablation index (AI) of 280–300, and the ablation duration was 10 s. Complete electrical isolation of the PLSVC successfully terminated the AF (Figure 2F,G). Following the procedure, intravenous isoproterenol was administered, and repeated electrophysiological tests with atrial pacing were conducted, none of which could induce any arrhythmias.

## 4 Outcome and Follow‐Up

After the ablation, isoproterenol infusion and atrial pacing were used to repeatedly verify that the patient did not experience AF. During and after the procedure, no complications occurred.

The patient was then followed up via telephone for 6 months, with monthly check‐ins. The follow‐up outcomes were defined as the patient experiencing palpitations accompanied by ECG‐confirmed AF, or the presence of AF lasting more than 30 s on a 24‐h Holter monitor, indicating a recurrence of AF. Regardless of the presence of palpitations, the patient underwent a 24‐h Holter monitor every 3 months.

Post‐procedure, the patient was prescribed amiodarone for 2 months and a novel oral anticoagulant (NOAC) for 3 months. At a follow‐up appointment 3 months later, no arrhythmias were detected, leading to the discontinuation of both amiodarone and the NOAC. During the entire follow‐up period, monthly phone calls and Holter monitoring showed no arrhythmias.

## 5 Discussion

AF is the most common cardiac arrhythmia, characterized by rapid and irregular contractions of the atrial myocardial cells. The incidence of AF increases with age and has been rising annually. It often occurs due to spontaneous depolarization of atrial tissue outside the sinoatrial node, primarily due to ectopic activity that commonly originates from the pulmonary veins (95%) and, less frequently, from the inferior and superior vena cava (5%) [3]. Rarely, it arises from abnormal structures such as the PLSVC [4, 5]. In terms of treatment, rhythm control is preferred for patients with concomitant heart failure or severe symptoms [6, 7].

PLSVC is a common congenital venous anomaly, also known as bilateral superior vena cava. Normally, the left superior vena cava regresses to form the ligament of Marshall. However, in approximately 0.3%–0.5% [8, 9] of the population, this regression does not occur, resulting in the formation of PLSVC. Most PLSVC cases drain into the right atrium via the CS, while in some patients, the right superior vena cava is atretic, and in very rare cases, the PLSVC drains into the left atrium [10] (Figure 2H–K).

Studies have shown that in patients with AF combined with PLSVC, 68.8% of the PLSVC can act as an ectopic trigger or maintenance substrate for AF, necessitating electrical isolation of the left superior vena cava. Additionally, 44.4% of these patients may have other types of arrhythmias, including atrial flutter, atrioventricular nodal reentrant tachycardia(AVNRT), and junctional tachycardia [10].

The mechanism of arrhythmias in PLSVC is complex and may be related to residual tissues from cardiac development during embryogenesis. During embryonic development, the primitive heart tube exhibits autonomic electrical activity, initially occurring in the sinus horn and main veins. As the heart matures, the pacemaker function gradually shifts to the sinoatrial node in the right heart. However, in some cases, such as with PLSVC, the original pacemaker tissue may not completely regress and remains in the PLSVC. These residual pacemaker tissues can cause electrophysiological abnormalities, particularly at the junction between the PLSVC and the CS. This structural abnormality can form ectopic trigger points, thereby initiating and maintaining AF. Additionally, overlapping myocardial sleeves in the PLSVC can also be a potential source of abnormal electrical activity and arrhythmias [10, 11].

Different scholars have varied strategies for the ablation of PLSVC. Successful catheter ablation has been reported at the proximal, mid, and distal segments of the PLSVC [2]. Regarding ablation power settings, some researchers have used a maximum power control mode of 65°C and 30W with a flow rate of 30 mL/min, while others have suggested using a power of 15–20W for ablation within the PLSVC, with each ablation point limited to a maximum duration of 20 s and a flow rate of 17 mL/min, targeting a maximum temperature of 43°C [11]. Additionally, some scholars have employed cryoablation, which has also been successful in eliminating AF [12].

During the procedure, it is crucial to consider the anatomical proximity of surrounding tissues. The left phrenic nerve descends along the anterolateral side of the PLSVC, extending down to the pericardium at the obtuse margin of the left ventricle [13]. Therefore, when performing ablation on the anterolateral side of the PLSVC, phrenic nerve pacing should be employed to avoid nerve damage.

In this case report, the patient was initially admitted with persistent AF. The preoperative echocardiogram showed a left atrial end‐systolic diameter (LA) of 50 mm and an EF of 33%. After 9 months of rhythm control to maintain sinus rhythm, a follow‐up echocardiogram revealed a reduction in heart size and normalization of the EF, further confirming that rhythm control is the preferred treatment for AF patients with heart failure to improve cardiac function.

Additionally, atrial substrate mapping identified the PLSVC as the trigger for paroxysmal AF, further confirming that AF can originate from abnormal structures. For ablation, the strategy involved targeting the fragmented potentials at the proximal PLSVC with an AI index of 280–300 and performing a 10‐s ablation. Under the protection of phrenic nerve pacing, electrical isolation of the PLSVC during AF successfully terminated the tachycardia, providing an effective treatment strategy for arrhythmias originating from similar anatomical anomalies. These findings emphasize the importance of considering PLSVC in the diagnosis and treatment of AF and demonstrate an effective ablation strategy for arrhythmias originating from similar anatomical anomalies, potentially influencing clinical practice.

## Author Contributions

**Shuang Zhang:** data curation, investigation, writing – original draft. **Mingxian Chen:** formal analysis, project administration, resources, supervision, writing – review and editing. **Yichao Xiao:** formal analysis, visualization. **Lin Hu:** project administration, visualization. **Hanze Tang:** investigation, visualization. **Liyi Liao:** data curation, resources. **Xuping Li:** conceptualization, funding acquisition, project administration, supervision, writing – review and editing.

## Ethics Statement

The authors have nothing to report.

## Consent

Written informed consent was obtained from the patient to publish this report in accordance with the journal's patient consent policy.

## Conflicts of Interest

The authors declare no conflicts of interest.

## Data Availability

The data that support the findings of this study are available from the corresponding author upon reasonable request.

## References

[1] S. K. Goyal , S. R. Punnam , G. Verma , and F. L. Ruberg , “Persistent Left Superior Vena Cava: A Case Report and Review of Literature,” Cardiovascular Ultrasound 6 (2008): 50, 10.1186/1476-7120-6-50. 18847480 PMC2576163

[2] M. Gao , Y. Bian , L. Huang , et al., “Catheter Ablation for Atrial Fibrillation in Patients With Persistent Left Superior Vena Cava: Case Series and Systematic Review,” Frontiers in Cardiovascular Medicine 9 (2022): 1015540, 10.3389/fcvm.2022.1015540. 36337869 PMC9632661

[3] B. Brundel , X. Ai , M. T. Hills , M. F. Kuipers , G. Y. H. Lip , and N. M. S. de Groot , “Atrial Fibrillation,” Nature Reviews. Disease Primers 8, no. 1 (2022): 21, 10.1038/s41572-022-00347-9. 35393446

[4] H. Liu , K. T. Lim , C. Murray , and R. Weerasooriya , “Electrogram‐Guided Isolation of the Left Superior Vena Cava for Treatment of Atrial Fibrillation,” Europace 9, no. 9 (2007): 775–780, 10.1093/europace/eum118. 17557767

[5] L. F. Hsu , P. Jaïs , D. Keane , et al., “Atrial Fibrillation Originating From Persistent Left Superior Vena Cava,” Circulation 109, no. 7 (2004): 828–832, 10.1161/01.Cir.0000116753.56467.Bc. 14757689

[6] P. Zimetbaum , “Atrial Fibrillation,” Annals of Internal Medicine 166, no. 5 (2017): Itc33–itc48, 10.7326/aitc201703070. 28265666

[7] P. Calvert , J. M. Farinha , D. Gupta , M. Kahn , R. Proietti , and G. Y. H. Lip , “A Comparison of Medical Therapy and Ablation for Atrial Fibrillation in Patients With Heart Failure,” Expert Review of Cardiovascular Therapy 20, no. 3 (2022): 169–183, 10.1080/14779072.2022.2050695. 35255780

[8] S. Löbig , A. Seitz , M. Feuerstein , R. Bekeredjian , and H. Mahrholdt , “Varicose Cardiac Veins in a Case of Persistent Left Superior Vena Cava and Stenosis of the Coronary Sinus Ostium,” European Heart Journal. Cardiovascular Imaging 21, no. 7 (2020): 786, 10.1093/ehjci/jeaa029. 32142101

[9] T. Tak , E. Crouch , and G. B. Drake , “Persistent Left Superior Vena Cava: Incidence, Significance and Clinical Correlates,” International Journal of Cardiology 82, no. 1 (2002): 91–93, 10.1016/s0167-5273(01)00586-1. 11786168

[10] Y. G. Kim , S. Han , J. I. Choi , et al., “Impact of Persistent Left Superior Vena Cava on Radiofrequency Catheter Ablation in Patients With Atrial Fibrillation,” Europace 21, no. 12 (2019): 1824–1832, 10.1093/europace/euz254. 31578551

[11] K. Higuchi , S. Iwai , Y. Yokoyama , and K. Hirao , “Persistent Left Superior Vena Cava as a Perpetuator of Atrial Fibrillation: Frequency Analysis Using Continuous Wavelet Transform Analysis,” Journal of Cardiovascular Electrophysiology 30, no. 9 (2019): 1701–1705, 10.1111/jce.14004. 31165514

[12] M. A. Schneider , A. Schade , M. L. Koller , and B. Schumacher , “Cryoballoon Ablation of Paroxysmal Atrial Fibrillation Within the Dilated Coronary Sinus in a Case of Persistent Left Superior Vena Cava,” Europace 11, no. 10 (2009): 1387–1389, 10.1093/europace/eup203. 19648587

[13] J. Peltier , C. Destrieux , J. Desme , C. Renard , A. Remond , and S. Velut , “The Persistent Left Superior Vena Cava: Anatomical Study, Pathogenesis and Clinical Considerations,” Surgical and Radiologic Anatomy 28, no. 2 (2006): 206–210, 10.1007/s00276-005-0067-7. 16402153

